# Referral patterns and proximity to palliative care inpatient services by level of socio-economic disadvantage. A national study using spatial analysis

**DOI:** 10.1186/1472-6963-12-424

**Published:** 2012-11-23

**Authors:** David C Currow, Samuel Allingham, Sonia Bird, Patsy Yates, Joanne Lewis, James Dawber, Kathy Eagar

**Affiliations:** 1 Discipline, Palliative and Supportive Services, Flinders University, 700 Goodwood Rd, Daw Park, South Australia, 5041, Australia; 2 Centre for Health Service Development, University of Wollongong, Wollongong, Australia; 3 Faculty of Health Sciences, Queensland University of Technology, Herston, Brisbane, Australia; 4 University of Technology, Broadway, Sydney, New South Wales, Australia

**Keywords:** Palliative care, Socio-economic disadvantage, Service planning, Referral patterns

## Abstract

**Background:**

A range of health outcomes at a population level are related to differences in levels of social disadvantage. Understanding the impact of any such differences in palliative care is important. The aim of this study was to assess, by level of socio-economic disadvantage, referral patterns to specialist palliative care and proximity to inpatient services.

**Methods:**

All inpatient and community palliative care services nationally were geocoded (using postcode) to one nationally standardised measure of socio-economic deprivation – Socio-Economic Index for Areas (SEIFA; 2006 census data). Referral to palliative care services and characteristics of referrals were described through data collected routinely at clinical encounters. Inpatient location was measured from each person’s home postcode, and stratified by socio-economic disadvantage.

**Results:**

This study covered July – December 2009 with data from 10,064 patients. People from the highest SEIFA group (least disadvantaged) were significantly less likely to be referred to a specialist palliative care service, likely to be referred closer to death and to have more episodes of inpatient care for longer time. Physical proximity of a person’s home to inpatient care showed a gradient with increasing distance by decreasing levels of socio-economic advantage.

**Conclusion:**

These data suggest that a simple relationship of low socioeconomic status and poor access to a referral-based specialty such as palliative care does not exist. Different patterns of referral and hence different patterns of care emerge.

## Background

Specialist palliative care services in Australia have grown rapidly over the last 20 years [1]. The site and model of service delivery have commonly developed in a way which is defined by local initiatives and have been *ad hoc*. A fundamental question arises as to whether there is evidence of discrepancies in referral to, or proximity to specialist palliative care services [1,2]. Are services distributed across the community in way that reflects the populations that they serve? [3] These concerns are supported by existing evidence for limited social and geographical access to palliative care services for certain groups in Australia [4].

Internationally, inequity in palliative care service provision has been demonstrated [5,6]. Previous research has suggested that lower socio-economic status has been associated with poorer utilisation of palliative care services [7]. Inequity in the distribution of palliative care services in disadvantaged areas [5] and increased travel times to specialist palliative care facilities from low socioeconomic areas [8] have been shown to further exacerbate disparities in care for people from lower socio-economic groups. Lower levels of palliative care service uptake by socioeconomically disadvantaged groups are considered to be driven by health and social service structures, and individual characteristics [9,10].

The relationship between the social determinants of health and well-being demands a commitment to address any inequitable access to services [11]. The foundations of Australia’s universal health care coverage, the established evidence for health service inequity and the vast geographical expanse of the continent confirms the need to ensure that utilisation of services is optimised for all people. To date, there are limited Australian data identifying any difference in access to palliative care services by socio-economic status.

Providing quality palliative care is not simply a social good. Emerging evidence demonstrates benefits to: patients (better symptom control, better *survival*, better adjustment to the disease process, better support); [12-22] caregivers (improved survival and adjustment having relinquished the role, better support in the role); [19,20,23-25] health services (fewer inpatient bed days, few admissions, lower overall costs); [21,26,27] and health practitioners (better support) [28]. These benefits then would suggest that there needs to be an assessment of referral to and proximity to services in order to ensure these benefits are available to all the people with the most complex end-of-life needs.

The use of linked population and healthcare data with geographic information (geocoding) has contributed to understanding service coverage [29-31]. The aims of this study are to examine:

i. Referral to specialist palliative care services by social-economic disadvantage and clinical phase [32]; and

ii. Proximity to, and utilization of inpatient services

## Methods

### Study setting

The Palliative Care Outcomes Collaboration (PCOC) is a national quality initiative to improve the clinical care of people with life-limiting illnesses. National coverage has grown rapidly since its inception in 2006 and, to date, more than 80% of all people seen by specialist palliative care services in Australia have data collected at point-of-care from referral until death. As a collateral opportunity, PCOC has allowed the identification of services that provide specialist palliative care nationally, including non-participating services.

Data are divided into three levels: patient data are collected once at the time of referral to the service; episode data are recorded each time the physical place of care changes; and phase data are coded with changes in clinical condition [32]. The clinical classification of ‘phase’ has four divisions relevant to this study: ‘stable’, ‘unstable’, ‘deteriorating’ and ‘terminal’. Independently of diagnosis, these descriptors reflect the last months of life and, in PCOC, are recorded at least weekly (for inpatient care) or with every clinical encounter (for community care) [32].

The Socio-Economic Index for Areas - Index of Disadvantage (SEIFA) based on data from the 2006 Australian Census is a summary measure of socio-economic conditions and is generated by the Australian Bureau of Statistics (ABS), and is updated after each census. The 2006 SEIFA score relates directly to the patient cohort reported here. This measure allocates a social disadvantage index to each postal area in Australia using census-derived variables including income, educational attainment, levels of employment and the number of households with a car [33]. In this study, the SEIFA index was collapsed into three SEIFA groups – low (most disadvantaged), medium and high (most advantaged) to represent three basic socio-economic levels. These three groups each represent approximately one third of the Australian population. The postcode of each patient’s residence could then be allocated to one of the three SEIFA groups.

To understand service utilisation, the proportion of referrals to specialist palliative care services from each SEIFA tertile and the clinical status (phase) at the start of palliative care episodes are compared.

To understand proximity to services, postcodes with geographical locators (longitude and latitude) allow mapping of spatial relationships. In this study, those spatial relationships were measured between the patients’ residences’ postcodes and specialist inpatient palliative care services’ postcodes if they received palliative care. Straight-line distances were calculated from a patient’s residence to the specialist palliative care service, allowing an estimate of proximity to services to be developed.

### Analyses

Chi-square, t-tests and analysis of variance (ANOVA) were used to compare the different measures of interest across the three SEIFA groupings as appropriate. Bonferroni corrections were applied as appropriate. A linear regression model fitted to log transformed distances was generated to adjust for the effect on SEIFA of age of the population who are dying.

Ethical oversight of this program is provided by the Human Research Ethics Committee of the University of Wollongong. This is a secondary analysis of de-identified aggregated patient and service data.

## Results

### Study data

The study used data from July 1 to December 31, 2009 collected at referral to specialist palliative care services across Australia. For geocoding and for socio-economic disadvantage, patient and service level postcodes data when first recorded were used in the analyses.

### Study setting

In total, 91 of the estimated 179 palliative care services that existed at this time participated in the PCOC data collection during the period. (Table 1) Fifty four percent of these services were in major cities, 32% in inner regional centres and 14% in outer regional or rural areas. While these 91 services represented 51% of palliative care services, it is estimated that they treated more than 80% of people referred to specialised palliative care services. These 91 services provided 751 (69.3%) of the estimated 1,084 specialist palliative care inpatient beds. Services provided various combinations of inpatient, consultative and community-based services (home visits and outpatient clinic appointments).

**Table 1:** Socio-Economic Index for Areas (2006 Census data; SEIFA) group for services that are and are not participating in the Australian Palliative Care Outcomes Collaboration (PCOC)

	**SEIFA group**		
	**Low**	**Medium**	**High**
Participating services (n=91)	47.3%	28.6%	24.2%
Non-participating services (n=88)	35.2%	37.5%	27.3%

### Patient population

In this period, 10,064 patients had data collected prospectively by PCOC. These people had 12,523 episodes of care (where an ‘episode’ changes each time the place of care changes). Overall, 54% of referrals were males (56% in the lowest SEIFA group; Table 2). The average age of the highest SEIFA group was significantly higher than the other two groups (p=0.002; Table 3). Eighty-two percent of those with a recorded diagnosis had cancer as their primary life-limiting illness and this proportion was the same for all three SEIFA groups (Table 2).

**Table 2:** Demographic and referral data at a patient level for all people in the Australian Palliative Care Outcomes Collaboration database July 1 – December 31, 2009, by Socio-Economic Index for Areas of Disadvantage (2006 Census data; SEIFA)

**Patient-level data**		**SEIFA Group**							
		**Low**^**2**^		**Medium**		**High**^**2**^		**Total**	
		**n**	**%**	**n**	**%**	**n**	**%**	**n**	**%**
Diagnosis	Cancer	2,781	83%	2,580	81%	1,961	82%	7,322	82%
	Non cancer	577	17%	614	19%	437	18%	1,628	18%
Gender	Male	2,114	56%	1,869	54%	1,447	51%	5,430	54%
	Female	1,630	44%	1,597	46%	1,396	49%	4,623	46%
Referral to specialist palliative care service^1^		3,751	37%	3,470	34%	2,843	28%	10,064	100%

^1^ Significant difference in SEIFA groups (Chi square: p < 0.001).

^2^ SEIFA low – most disadvantaged; SEIFA high – most advantaged.

**Table 3:** Demographic and proximity data at a patient level for all people in the Australian Palliative Care Outcomes Collaboration database July 1 – December 31, 2009, by Socio-Economic Index for Areas of Disadvantage (2006 Census data; SEIFA)

**Patient-level data**		**SEIFA Group**		
		**Low**^**4**^	**Medium**	**High**^**4**^
Age (years)	Mean^2^	70.3	70.1	71.3
	Median	72	72	73
	IQR^1^	19	20	20
Distance from inpatient care (kilometres)	Mean^3^	43.4	29.9	14.6
	Median	9.9	9.4	8.1
	IQR^1^	25.8	14.6	9.9

^1^ Inter quartile range.

^2^ High SEIFA group significantly older than both other groups (multiple t-test: largest p = 0.002).

^3^ Low SEIFA group significantly greater than both other groups (multiple t-test: largest p < 0.001).

^4^ SEIFA low – most disadvantaged; SEIFA high – most advantaged.

### Referral to, and care provided by specialist palliative care services by socio-economic status

Using Chi-square, the distribution of referrals to specialist palliative care services was significantly lower for people from the highest SEIFA group (lowest 3744 (37.2%), medium 3466 (34.5%) and highest 28.3%; p<0.001). This was also reflected in a significantly lower number of episodes of care provided. As a marker of timeliness of referral, people from the highest SEIFA group were also significantly less likely to be classified to the palliative care ‘stable’ phase (19.4% vs 22.6% (medium) and 24.2% (lowest); p=0.001-) [31]. (Table 4) Episodes of care were significantly more likely to be in the inpatient setting for people from the highest SEIFA group (71% compared with 63% for the other two groups; p<0.001) with significantly longer mean inpatient lengths of stay (highest 13.9 days; medium 11.6 days and lowest 11.5 days; p<−0.001 ) (Table 5).

**Table 4:** Episode of care level data in the Australian Palliative Care Outcomes Collaboration database July 1 – December 31, 2009, by Socio-Economic Index for Areas of Disadvantage (2006 Census data; SEIFA)

**Episode-level data**		**SEIFA group**							
		**Low**^**3**^		**Medium**		**High**^**3**^		**Total**	
		**n**	**%**	**n**	**%**	**n**	**%**	**n**	**%**
Place of care^1^	Inpatient	2,919	63%	2,871	63%	2,358	71%	8,148	65%
	Community	1,703	37%	1,693	37%	979	29%	4,375	35%
Phase at beginning of a patient’s palliative care episode	Stable^2^	1,076	24%	971	23%	620	19%	2,667	22%
	Unstable	1,786	40%	1,639	38%	1,405	44%	4,830	40%
	Deteriorating	1,246	28%	1,367	32%	960	30%	3,573	30%
	Terminal	343	8%	320	7%	219	7%	882	7%

(Episode changes every time physical place of care changes).

^1^ High SEIFA group significantly different from both other groups (multiple chi-square: largest p < 0.001).

^2^ High SEIFA group has a significantly lower proportion of episodes starting ‘Stable’(multiple chi-square; largest p = 0.001).

^3^ SEIFA low – most disadvantaged; SEIFA high – most advantaged.

**Table 5:** Episode of care level data in the Australian Palliative Care Outcomes Collaboration database July 1 – December 31, 2009, by Socio-Economic Index for Areas of Disadvantage (2006 Census data; SEIFA)

			**SEIFA group**		
**Episode-level data**			**Low**^**3**^	**Medium**	**High**^**3**^
Episode length (elapsed days)	Inpatient care^1^ (excluding day only admissions)	Mean	11.5	11.6	13.9
		Median	7	7	8
		IQR	12	12	14
		n	2,802	2,768	2,257
	Community care^2^ (including day only admissions)	Mean	46.9	42.9	41.8
		Median	2	19	21
		IQR	57	49	44
		n	1,233	1,355	792

(Episode changes every time physical place of care changes).

^1^High SEIFA group significantly different from both other groups (multiple t-test: largest p < 0.001).

^2^ High SEIFA group significantly different from both other groups (multiple t-test:

^3^ SEIFA low – most disadvantaged; SEIFA high – most advantaged.

### Proximity to inpatient services by socio-economic status

Using geocoding, for people who had at least one inpatient episode of care, there were significant differences between the proximity to that care by SEIFA. For the lowest group, distances were a mean of 43.4km compared to medium (29.9km) and highest (14.6km) (Table 3). Using a regression model with distance as the dependent variable, and controlling for SEIFA, rurality and age, the model demonstrated that older people were significantly more likely to be proximate to inpatient services (R^2^ 0.78; for age, p<0.001).

## Discussion

This study confirms that there are significant differences in the uptake of, and proximity to specialist palliative care services in Australia. These differences are associated with socio-economic status in a complex way. For the highest SEIFA category (those who were least disadvantaged) there was: lower uptake of specialist palliative care services; later in the course of the illness; with referral less likely to be in the stable phase; better proximity to inpatient services; and longer duration of inpatient care [34,35]. These data support previous population-based studies in the United States, Canada and Australia that observed disparities in access to specialist palliative care services by socio-economic disadvantage, although the patterns appear somewhat different to previous reports [36-38].

Not all studies on disparities on referral to specialist palliative care services have seen socio-economic differences [39]. Higher levels of community-based care in the lowest tertile shifts the responsibility of care to families and friends, including the financial cost of caring. Such differences in community-based care are amplified if the most advantaged SEIFA group are able to access a range of services including user-pays nursing and home care [34,40].

Proximity to specialist palliative care services may have significant implications for health service delivery more broadly. In an analysis of more than 28,000 people over the age of 65 who died of cancer within one year of diagnosis, while controlling for key demographic and clinical factors, physical proximity to hospice services was an independent predictor of less aggressive end-of-life care [41].

### Needs based services

Do people from lower socio-economic strata have different needs? There is no evidence that needs in the most disadvantaged communities are any less and, arguably, given the social deprivation experienced across the life span, needs will be greater [42]. This challenges directly hospice and palliative services to consider how to provide needs-based care that genuinely serves the whole population in order to achieve equitable *outcomes* (not simply equitable *access*) [34]. The patterns demonstrating higher levels of chronic illness and of earlier mortality for people from greater socio-economic disadvantage would suggest that there is an even greater focus required to ensure equitable outcomes at the end of life given, for example, the higher likelihood of dependent children given the age at which people are dying.

### Limitations

These data also assume that for each SEIFA group the likelihood of death is equal. The discrepancy in access is likely to be greater given that socio-economic status at the end of life is going to be lower for a significant proportion of the population given diminishing disposable income for older people. The services not represented in the patient-level data were more likely to be rural where greater socio-economic disadvantage is also likely. Discrepancies in the way services are utilised are magnified in subtle ways – the costs of community-based care are borne largely by the family and the proportion of care provided in the community is greatest in the most disadvantaged group in the community. Likewise, the costs of travel to inpatient units will be disproportionately borne by those least able to afford it.

### Limitations of the study (sample)

The study does not include all services in Australia, but the missing services tend to be smaller more rural services where poor proximity to care will add to disadvantage. Their inclusion would likely magnify the effects seen in the data presented. Coding has been done for the services that are missing, but the populations who access these services are not available in this data set.

People who already experience geographic isolation from services may simply never be referred and hence not represented in this sample further underestimating disparities. Only people who used designated inpatient palliative care beds form the basis of this analysis. This therefore underestimates the impact of distance for people who chose not to have inpatient care because the distances were too great, or had care at a hospital closer to home in a non-designated bed.

### Limitations of the study (data)

The current studies refer only to distance not ease of travel which incorporates distance, mode of travel, costs and other factors that contribute to accessibility. Although absolute distances may be much shorter in metropolitan areas, travel time especially when relying on public transport may still be very challenging [43].

Using straight-line distances between the patient’s postcode and the postcode of the inpatient facility, it is likely that the travel distance is underestimated for patients living in regional and rural areas. This is because postal areas are not uniform in geographic size. Hence, patients living in the same postcode as a palliative care service in a regional area (assigned a travel distance of 0) may need to travel much further than a patient living in a major city who lives in the same postcode as a palliative care service. While straight-line distance travelled is not an exact measure of travel burden, it acts as a strong indicative measure of access.

### Generalisability

These data refer to the Australian health and social systems, thus limiting generalisability to other settings. Further, there are differential rates of coverage for the services represented: 51% of all services; 69% of all designated inpatient beds; and approximately 80% of all patients referred to specialist palliative care services in the country. As noted in *Limitations*, this therefore probably underestimates the differences seen for the most socially disadvantaged rural patients.

### Future research directions

Research in the future should look specifically at needs, complexity of care and outcomes at the end of life by socio-economic status. Given that one in two people with an expected death will *not* access palliative care in Australia, population level studies are required to ensure that service delivery better incorporates needs-based access to specialist palliative care services across the community [44]. Accessibility to services needs to be geocoded for *all* predictable deaths accepting that only a sub-set would derive benefit from referral to specialised palliative care services.

### Conclusion

Most palliative care services have had little jurisdictional or local planning. With few exceptions, this ‘organic’ process has been built around community interests and goodwill as well as the availability of existing hospital stock rather than sound principals of health service planning. Population-based service planning may require further affirmative action to overcome some of the geographical difficulties highlighted by this current study.

Better use of new models of care with emerging technologies such as video-conferencing for clinical consultations and family conferences may relieve some burden related to poor proximity to services. This becomes an overwhelming imperative for areas where long distances need to be travelled by caregivers, families and friends in metropolitan peri-urban and rural areas when inpatient services are dislocated from that person’s own environment.

Inpatient care needs to be accessible since much of it is required at short notice due to unexpected changes in condition. Inpatient care also needs to be physically proximate so that family and friends can continue to support patients.

## Competing interests

The author(s) declare that they have no competing interests.

## Authors’ contributions

Conception and design DC,KE; Planning DC,KE,PY; data acquisition DC,PY,JL,KE; analysis SA,SB,JD; interpretation KE,PY,JL,DC; Drafting DC, Critical Revision DC,SA,SB,PY,JL,JD,KE. All authors read and approval the final manuscript.

## Pre-publication history

The pre-publication history for this paper can be accessed here:

http://www.biomedcentral.com/1472-6963/12/424/prepub
